# Searching Optimum Self-Brazing Powder Mixtures Intended for Use in Powder Metallurgy Diamond Tools—A Statistical Approach

**DOI:** 10.3390/ma18122726

**Published:** 2025-06-10

**Authors:** Andrzej Romański, Piotr Matusiewicz, Elżbieta Cygan-Bączek

**Affiliations:** 1 Faculty of Metals Engineering and Industrial Computer Science, AGH University of Kraków, al. A. Mickiewicza 30, 30-059 Kraków, Poland; matus@agh.edu.pl; 2 Centre of Powder and Composite Materials, Łukasiewicz Research Network-Institute of Non-Ferrous Metals, ul. Sowińskiego 5, 44-100 Gliwice, Poland; elzbieta.cygan-baczek@imn.lukasiewicz.gov.pl

**Keywords:** diamond impregnated tools, wire saw, self-brazing powders, ANOVA

## Abstract

This paper presents a study on optimising self-brazing powder mixtures for powder metallurgy diamond tools, specifically focusing on wire saws used in cutting natural stone. The research aimed to understand the relationship between the chemical composition of powder mixtures and the hardness of the sintered matrix. The experimental process involved the use of various commercially available powders, including carbonyl iron, carbonyl nickel, atomised bronze, atomised copper, and ferrophosphorus. The samples made of different powder mixtures were compacted and sintered and then characterised by dimensional change, density, porosity, and hardness. The obtained results were statistically analysed using an analysis of variance (ANOVA) tool to create linear regression models that relate the material properties to their chemical composition. The investigated materials exhibited excellent sintering behaviour and very low porosity, which are beneficial for diamond retention. Very good sinterability of powder mixtures can be achieved by tin bronze addition, which provides a sufficient content of the liquid phase and promotes the shrinkage during sintering. Statistical analysis revealed that hardness was primarily affected by phosphorous content, with nickel having a lesser but still significant impact. The statistical model can predict the hardness of the matrix based on its chemical composition. This model, with a determination coefficient of approximately 80%, can be valuable for developing new metal matrices for diamond-impregnated tools, particularly for wire saw beads production.

## 1. Introduction

Powder metallurgy diamond tools are widely used for cutting and machining natural stones, reinforced concrete, and hard-to-cut ceramic materials, such as glass, cemented carbides, and others. A common feature of all metal–diamond impregnated tools is diamond grits embedded in the metallic matrix, which holds diamond particles firmly and erodes at rates comparable to diamond loss.

For years, as a matrix for powder metallurgy diamond tools, different types of cobalt and cobalt-base powders have been used, mainly because of very good mechanical properties in the as-consolidated state and their wear resistance adequate to the workpiece’s abrasiveness [1,2]. Cobalt powders are an excellent choice for diamond-impregnated segments produced by the hot pressing technique but, for a standard powder metallurgy route, that is, cold pressing and subsequently sintering, show some limitations. The main disadvantage of the cobalt matrix is the necessity of using brazing alloys to increase the bonding between the diamond containing layer and the steel shanks or sleeves [3]. To avoid this additional treatment, that is brazing, the industry has started to substitute cobalt with cheaper powders, which preferably exhibit a self-brazing capability at the sintering temperature. These powders are designed to improve the bonding strength between the diamond and matrix as well as between the diamond-impregnated layer and the steel support of the segments, thereby increasing the wear resistance and cutting efficiency of the tool [4,5]. Recent advancements in the development of self-brazing powders for sintered diamond tools have focused on improving the properties of the powders, such as enhancing their low-temperature sintering characteristics, wear resistance, physical and mechanical characteristics, and bonding strength [6,7,8].

The resistance of the matrix to wear must be carefully selected depending on the abrasiveness of the workpiece. Machining easy-to-cut stones (sandstones) usually requires a matrix of higher resistance to wear, owing to the extensive wear caused by the slurry produced during cutting, which leads to a decrease in tool performance and lifespan. On the other hand, the use of a matrix characterised by a lower resistance to wear is usually sufficient for the effective treatment of hard-to-cut materials. In the sintered diamond tools industry, the measurement of wear resistance is not a common practice because it requires dedicated (often very expensive) devices. Hence, hardness becomes a crucial parameter for quality control of the sintered segments and their resistance to the wear estimator. By performing hardness measurements, the produced segments can be classified according to their potential wear behaviour. It is evident that the hardness of the matrix can be modified by the addition of alloying elements to the base powder. For mixtures based on iron, commonly used additives are nickel, phosphorous (in the form of Fe_3_P, Fe_2_P, or ferrophosphorus containing ~10% P), copper, and tin bronzes [9,10,11,12]. The addition of tin bronzes (usually containing 10–20% Sn) is required to provide a sufficient amount of liquid phase during sintering, which makes the powder mixture self-brazing. To modify the hardness of a matrix made of a mixture of different starting powders in a conscious manner, it is necessary to know how alloying elements affect the hardness of a complex system.

Analysis of variance (ANOVA) is a powerful statistical tool widely used in materials engineering to evaluate the significance of various process parameters and their interactions on final product properties. In powder metallurgy, ANOVA helps determine which factors, such as particle size distribution, compaction pressure, sintering temperature, and composition, have the most significant impact on the mechanical, physical, and microstructural characteristics of the final component [13,14,15,16,17]. In this study, ANOVA was utilised to understand the relationship between the chemical composition of the powder mixtures showing self-brazing characteristics and the properties of the sintered matrix intended for use in powder metallurgy diamond tools, mainly focusing on its hardness.

## 2. Experimental

The following commercially available powders were used to produce the mixtures:

(a) Carbonyl iron (FSSS = 6.5 μm);
(b) Carbonyl nickel (grade T123, FSSS = 5.4 μm);
(c) Atomised bronze containing 20% (B20, particle size < 53 μm), 15% (B15, particle size < 45 μm), and 10% (B10, particle size < 45 μm) of tin;
(d) Atomised copper (grade LT16, particle size < 45 μm);
(e) Carbonyl ferrophosphorus containing 9% of P (Fe-P, D50 = 6 μm);
(f) Ferrophosphorus containing 15.6% of P (Fe_3_P, D50 = 8 μm).

**Figure 1:**
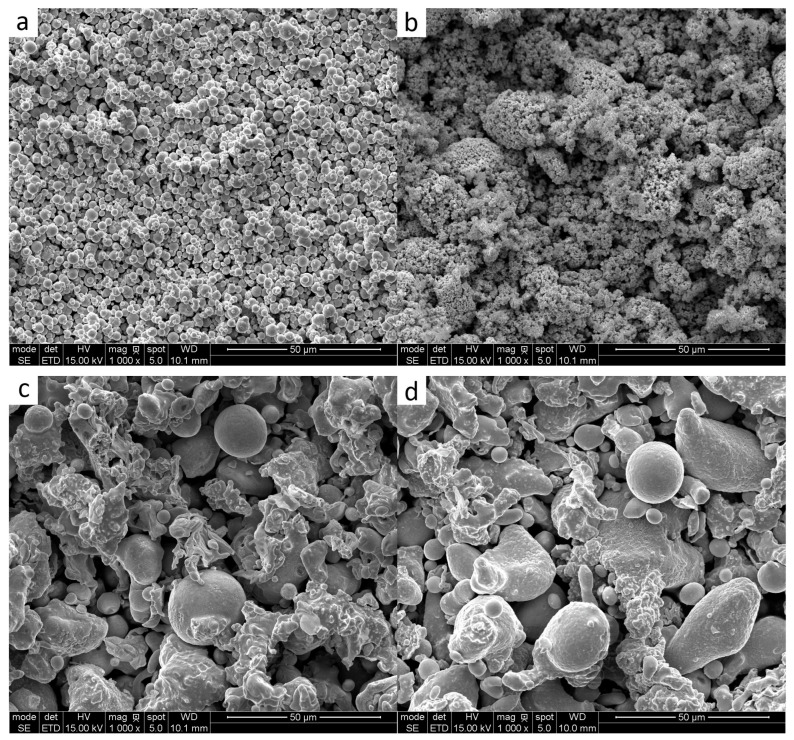

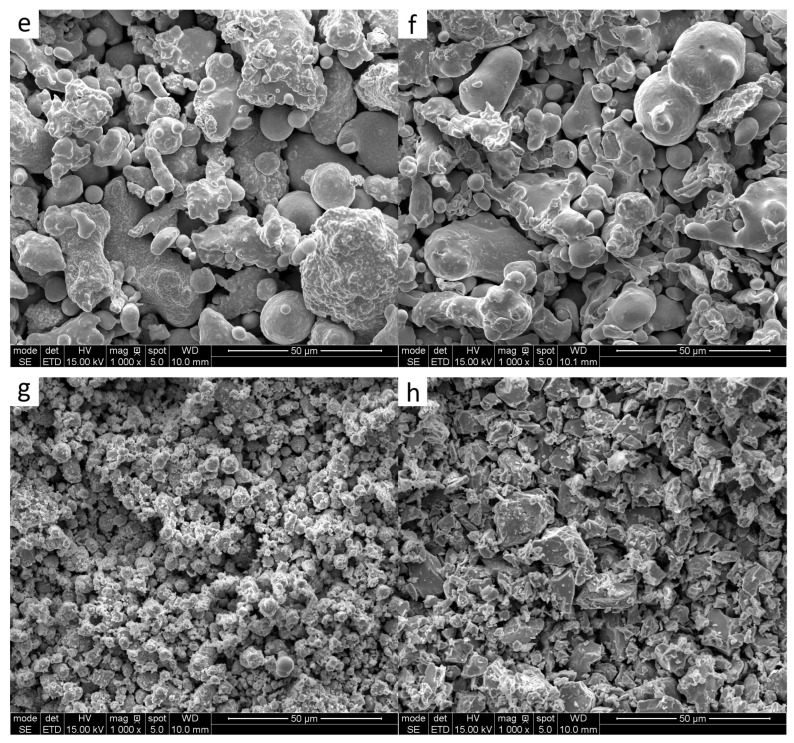
Raw materials used in the research: iron (**a**), nickel (**b**), bronze B20 (**c**), bronze B15 (**d**), bronze B10 (**e**), copper (**f**), Fe-P (**g**), and Fe_3_P (**h**).

The morphologies of the individual powders are shown in Figure 1.

All the powders were provided by MC Diam, Sandomierz—a Polish manufacturer of sintered diamond-impregnated tools. One of the main criteria for preparing the powder mixtures was to ensure a sufficient amount of liquid phase during sintering in order to obtain a self-brazing capability of the mixture by varying the content of tin bronze. The hardness of the as-sintered materials was modified mainly by different additions of phosphorous in the form of ferrophosphorous Fe-P or Fe_3_P and, in a limited way, by the amount of nickel. The compositions of the investigated mixtures are listed in Table 1 in increasing order of the iron content.

Pure powder mixtures were used to avoid any effect of the lubricant on the properties of the as-sintered specimens. Compacts 15 × 15 × 5 mm, approximately 5 g in mass each, were produced by cold compaction in a rigid die using a single-action press under a pressure of 260 MPa, which is typical in the powder metallurgy diamond tools industry. All compacted specimens were sintered in a laboratory tube furnace at 950 °C for 30 min in flowing pure hydrogen. The heating rate was set at 15 K/min, whereas cooling to 650 °C was performed in a furnace and then in a cooling zone. This method allows for the simulation of real conditions of the cooling rate in an industry belt furnace that operates in the MC Diam factory. The as-sintered samples were measured to the nearest 0.01 mm to calculate their shrinkage as one of the quality parameters. The densities of the as-sintered specimens were evaluated using Archimedes’ principle [18]. In addition, hardness measurements were performed using a Vickers indenter under a load of 9.81 N (1 kgf). As raw materials can formulate different types of solid solutions, accurate calculation of the theoretical density of the investigated materials is impossible. For this reason, the porosity of the as-sintered samples was calculated by the optical method on three different micrographs captured at 100× magnification using the DeltaOptical DLTCamViewer software (ver. x64, 4.11.20805.20220506). The cross-section preparation includes grinding on #220 SiC paper and polishing using 9, 3, and 1 μm diamond polishing suspensions. The final polishing was carried out with colloidal silica suspension. In order to avoid any negative effects on pore size, the polished cross-sections were not etched.

**Figure 2:**
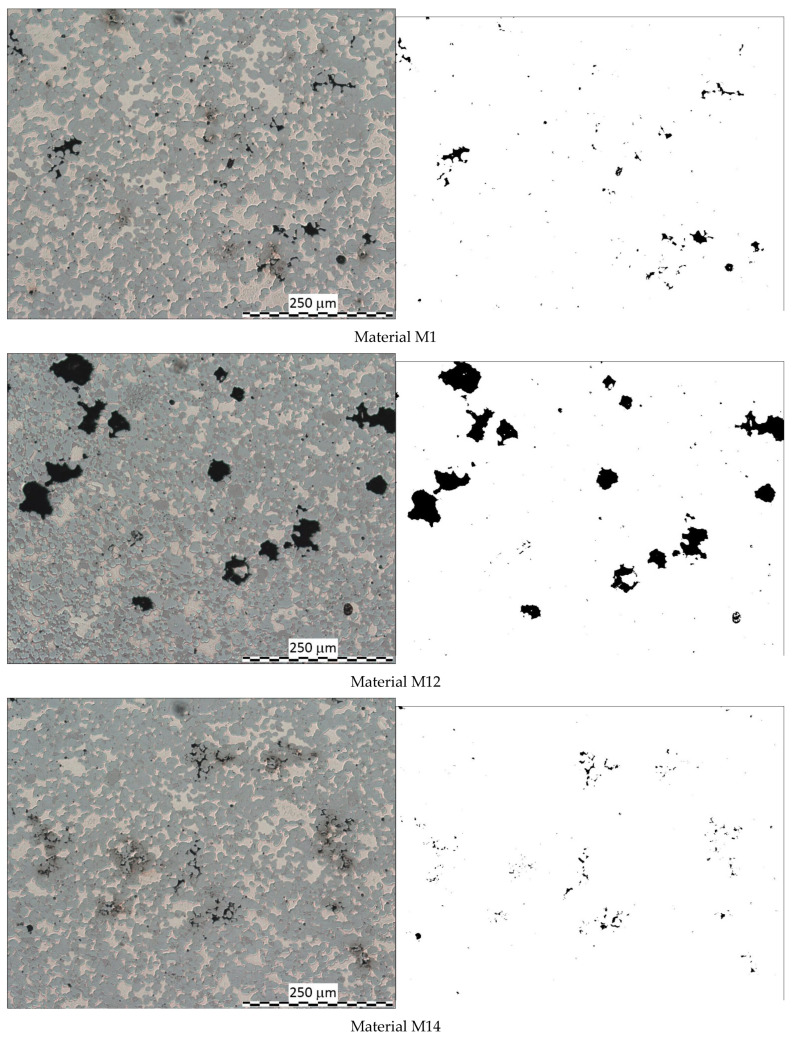
Example of microstructure and its binarized image of selected materials.

First, a representative micrograph of the microstructure was subjected to binarization, and the black pixels representing the pores were counted. Each time, the binarization filter was set to obtain the best mapping of the pores in comparison to the original micrograph. The selected typical microstructures and their binarized images are shown in Figure 2.

Porosity was calculated as the fraction of the number of black pixels to the total number of pixels in the micrograph. The procedure described above was applied to calculate the porosity of all investigated materials. The obtained data were statistically evaluated using the commercial software Statistica (v. 13.3) by the analysis of variance tool (ANOVA) to create a statistical model of the dependence between the chemical composition of the mixtures and the hardness, shrinkage, and porosity of the as-sintered materials.

## 3. Results

The dimensional change, density, porosity, and Vickers hardness values are summarised in Table 2.

**Figure 3:**
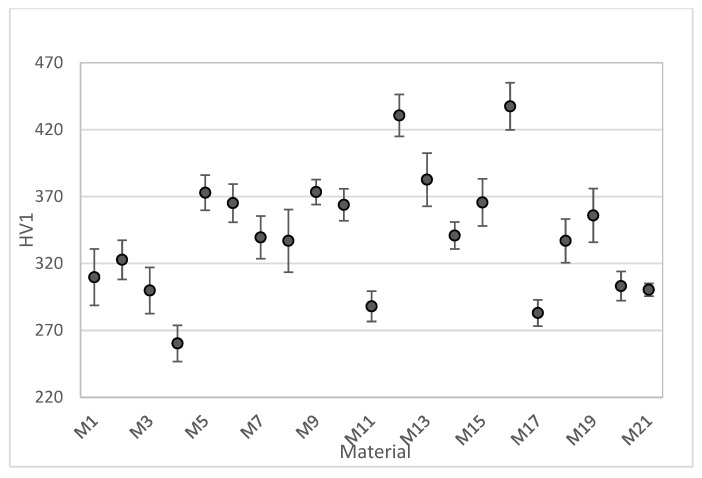
Confidence intervals for the mean hardness of tested materials.

Figure 3 shows the mean hardness and standard deviations of all investigated materials. As can be seen, there is a significant difference in hardness between specimens. The maximum value (437 HV1) was recorded for the M16 material, whereas the minimum value (260 HV1) was recorded for the M4 material.

All hardness values were used to create a multiple linear regression model to estimate the relationship between the quantitative dependent variable, hardness, and independent variables, Cu, Sn, Ni, and P contents. The equation is as follows:

Hardness HV1 = 264 − 1.38 [%Cu] + 6.29 [%Sn] + 5.99 [%Ni] + 29 [%P](1)

The standard error of the estimation is 22.27. To verify the significance of the above model, Fisher’s exact test was performed (Table 3). As the *p*-value is less than the statistical significance level α = 0.05, it can be assumed that there is a linear relationship between the dependent variable, hardness, and the independent variables (i.e. copper, tin, nickel, and phosphorous contents), for which the determination coefficient R^2^ is equal to 81.1%.

Based on the data calculated from the multiple regression model, which included four independent variables, Equation (1) was modified by removing one of the independent variables for which the highest *p*-value was calculated, which is tin content. Thus, a new, modified equation is as follows:

Hardness HV1 = 295.6 − 1.63 [%Cu] + 5.36 [%Ni] + 36.66 [%P](2)

The standard error of the estimation is 22.86. As the *p*-value is less than 0.05, it indicates that the model terms are significant, with a determination coefficient R^2^ of 78.8—Table 4.

**Figure 4:**
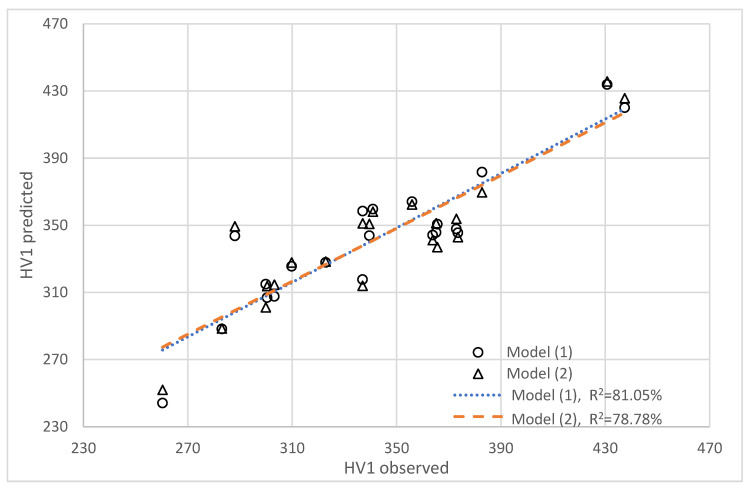
Predicted hardness based on models (1) and (2) compared to observed values.

Figure 4 shows the verification of the above models with experimental data. As can be seen, there is a minor difference between the observed values and models (1) and (2), for which the determination coefficient R^2^ is 81.1% and 78.8%, respectively.

A similar procedure was applied to establish the relationship between the dimensional change ΔL/L_0_ and the density of the investigated materials. Interestingly, a multiple regression model that includes four independent variables indicates that a linear dependence between both ΔL/L_0_ or porosity and Cu, Sn, Ni, and P contents is statistically insignificant for the assumed confidence level. Detailed ANOVA data are presented in Table 5 and Table 6.

## 4. Discussion

The investigated materials exhibited very good sintering behaviour that could be seen when the dimensional changes were analysed. The maximum shrinkage (−12.81%) was recorded for the M3 material, and the minimum shrinkage (−10.81%) was recorded for the M12 material. The excellent sinterability of the prepared powder mixture had a significant impact on the porosity of the as-sintered materials. With the exception of the M12 material, the porosity varied from 1.60% to 0.28%. Such low porosity should ensure that the matrix has superior retention properties to firmly hold diamond crystals during the machining of the workpiece. From this point of view, almost all investigated materials can potentially be used as a matrix in diamond-impregnated tools, especially in the production of wire saw beads. Unexpectedly, the statistical analysis of the dimensional change and porosity showed no linear relationship between these values and the chemical composition of the investigated materials. This is probably due to the sufficient liquid phase content that occurs during sintering and excellent wetting conditions, leading to a near pore-free microstructure, and, in addition, to the self-brazing capabilities of the investigated powder mixtures. Moreover, the use of fine powders promotes the sinterability of the tested materials and helps to reduce their porosity. In this research, carbonyl powders are mainly used as a base material, and the finer the powder, the better its sinterability, which is a well-known rule.

In powder metallurgy diamond tools, hardness is often used as an estimator of the potential resistance of the matrix to wear. In general, the harder the matrix, the higher its resistance to abrasive wear. Of course, there are some exceptions to this rule, especially when composites reinforced with dispersed ceramic phases are considered or strain-induced phase transformation occurs in the matrix during machining. In this study, no ceramic particles were used as a reinforcement, and no effects of strain-induced phase transformation were observed, which is why the hardness of the tested materials could be associated with their wear resistance. As previously mentioned, the investigated matrices were characterised by hardness from 260 HV1 to 437 HV1. This means that the potential resistance of these materials to wear can be modified according to the specific application of a tool. For cutting easy-to-cut workpieces such as sandstones, it is better to use a matrix with a high resistance to wear, while when machining difficult-to-cut materials (granite), it is preferable that the matrix is characterised by lower resistance to wear. This ensures the self-sharpening effect of the tool.

A statistical analysis of hardness allows for the establishment of a linear relationship between this property and the chemical composition of the material. In the first approach, the contents of all the alloying elements (Cu, Sn, Ni, P) were considered. It is evident from Equation (1) that the hardness of the material is mainly affected by the phosphorous content, whereas the effect of nickel is nearly five times weaker than that of phosphorous. As the tin content is dependent on the amount of bronze applied in the experiment (only one material contains bronze B10, eight materials—bronze B15, and twelve materials—bronze B20), this probably leads to the maximum *p*-value for tin, which exceeds the statistical significance level. By removing the tin content from model (1), the linear relationship between hardness and Cu, Ni, and P contents is given by Equation (2). It was confirmed that hardness is mostly dependent on phosphorous, and its effect is almost seven times stronger than that of nickel. The standard errors of estimation of models (1) and (2) are quite the same, which proves the high accuracy of the created models, for which the calculated determination coefficients R^2^ are 81% and 79%, respectively. The created models can be helpful in developing a new metal matrix for diamond-impregnated tools, especially in wire saw beads production, and in making conscious decisions regarding the chemical composition of the material.

## Figures and Tables

**Table 1:** Composition of powder mixtures (all values in wt.%).

No.	Mixture	Mixture Composition								Chemical Composition				
		FeCN	Fe-P	Fe_3_P	B20	B15	B10	Ni	Cu	Fe	Cu	Sn	Ni	P
M1	31FeCN + 25B15 + 15FeP + 20Cu + 9Ni	31	15	-	-	25	-	-	20	44.6	41.3	3.8	9.0	1.3
M2	31FeCN + 27B15 + 18Cu + 15FeP + 9Ni	31	15	-	-	27	-	-	18	44.6	41.0	4.1	9.0	1.3
M3	40B20 + 27.6FeCN + 22.4FeP + 10Cu	27.6	22.4	-	40	-	-	-	10	48.0	42.0	8.0	-	2.0
M4	40FeCN + 30Cu + 20B15 + 10FeP	40	10	-	-	20	-	-	30	49.1	47.0	3.0	-	0.9
M5	39FeCN + 20B20 + 20Cu + 12Fe3P + 9Ni	39	-	12	20	-	-	9	20	49.1	36.0	4.0	9.0	1.9
M6	31FeCN + 20B20 + 20FeP + 20Cu + 9Ni	31	20	-	20	-	-	9	20	49.2	36.0	4.0	9.0	1.8
M7	31FeCN + 25B15 + 20FeP + 15Cu + 9Ni	31	20	-	-	25	-	9	15	49.2	36.3	3.8	9.0	1.8
M8	40B15 + 32FeCN + 19FeP + 9Ni	32	19	-	-	40	-	9	-	49.3	34.0	6.0	9.0	1.7
M9	34FeCN + 25B20 + 17FeP + 15Cu + 9Ni	34	17	-	25	-	-	9	15	49.5	35.0	5.0	9.0	1.5
M10	41.5FeCN + 25B20 + 15Cu + 9.5Fe3P + 9Ni	41.5	-	9.5	25	-	-	9	15	49.5	35.0	5.0	9.0	1.5
M11	39B10 + 33FeCN + 19FeP + 9Ni	33	19	-	-	-	39	9	-	50.3	35.1	3.9	9.0	1.7
M12	40FeP + 35B20 + 15FeCN + 10Ni	15	40	-	35	-	-	10	-	51.4	28.0	7.0	10.0	3.6
M13	35FeCN + 35B20 + 20FeP + 10Ni	35	20	-	35	-	-	10	-	53.2	28.0	7.0	10.0	1.8
M14	37FeCN + 35B15 + 19FeP + 9Ni	37	19	-	-	35	-	9	-	54.3	29.8	5.3	9.0	1.7
M15	43FeCN + 35B20 + 15FeP + 7Ni	43	15	-	35	-	-	7	-	56.7	28.0	7.0	7.0	1.3
M16	35FeP + 30B20 + 25FeCN + 10Ni	25	35	-	30	-	-	10	-	56.9	24.0	6.0	10.0	3.2
M17	45FeCN + 35B15 + 15FeP + 5Cu	45	15	-	-	35	-	-	5	58.7	34.8	5.3	-	1.3
M18	53FeCN + 35B15 + 7Fe3P + 5Ni	53	-	7	-	35	-	5	-	58.9	29.8	5.3	5.0	1.1
M19	57FeCN + 25B20 + 10Ni + 8Fe3P	57	-	8	25	-	-	10	-	63.8	20.0	5.0	10.0	1.2
M20	65.5FeCN + 20B20 + 6.5Fe3P + 5Cu + 3Ni	65.5	-	6.5	20	-	-	3	5	71.0	21.0	4.0	3.0	1.0
M21	61FeCN = 20B20 + 11FeP + 5Cu + 3Ni	61	11	-	20	-	-	3	5	71.0	21.0	4.0	3.0	1.0

**Table 2:** Material characteristics.

Material No.	Dimensional Change DL/L0 %	Density g/cm^3^	Porosity % *	HV1 **
M1	−11.44	8.15	1.46	310 ± 67
M2	−11.67	8.14	1.60	323 ± 46
M3	−12.81	8.18	1.19	300 ± 55
M4	−11.45	8.16	1.10	260 ± 43
M5	−11.42	7.98	0.79	373 ± 41
M6	−11.91	8.14	1.13	365 ± 45
M7	−11.49	8.12	0.40	340 ± 50
M8	−11.89	8.10	0.71	337 ± 74
M9	−12.35	8.11	0.60	374 ± 29
M10	−11.61	8.01	0.57	364 ± 38
M11	−11.49	8.06	0.37	288 ± 36
M12	−10.81	7.20	7.58	431 ± 50
M13	−11.21	7.98	1.21	383 ± 63
M14	−11.82	8.04	0.28	341 ± 32
M15	−10.94	8.07	0.32	366 ± 56
M16	−12.12	7.93	0.65	437 ± 56
M17	−12.29	8.02	1.38	283 ± 31
M18	−10.89	7.98	0.48	337 ± 52
M19	−11.25	7.87	1.30	356 ± 63
M20	−11.82	7.89	0.69	303 ± 35
M21	−12.20	8.04	0.80	300 ± 15

*—mean value from 3 micrographs, **—mean value from 10 measuring points, scatter intervals estimated at 95% confidence level.

**Table 3:** Summary of multiple regression model.

N = 21	Standardised Regression Coefficient Beta	Std. Error of Beta	Regression Coefficient b	Std. Error of b	*t* Test Value t(16)	*p*-Value
Intercept			263.9546	36.6013	7.2116	0.000002
% Cu	−0.2220	0.1149	−1.3796	0.7139	−1.9325	0.071193
% Sn	0.1848	0.1333	6.2908	4.5399	1.3869	0.184853
% Ni	0.4711	0.1278	5.9912	1.6257	3.6852	0.002004
% P	0.4142	0.1443	29.0141	10.1091	2.8701	0.011109

Correlation coefficient R = 0.9003; Determination coefficient R^2^ = 0.8105; Adjusted determination coefficient R^2^adj = 0.7632; F test value F(4, 16) = 17.114; *p*-value < 0.00001; Std. error of estimation = 22.27.

**Table 4:** ANOVA for hardness (tin content excluded).

N = 21	Standardised Regression Coefficient Beta	Std. Error of Beta	Regression Coefficient b	Std. Error of b	*t* Test Value t(16)	*p*-Value
Intercept			295.6330	29.3471	10.0737	0.000000
% Cu	−0.2621	0.1141	−1.6291	0.7093	−2.2969	0.034597
% Ni	0.4217	0.1260	5.3627	1.6029	3.3457	0.003833
% P	0.5233	0.1242	36.6556	8.6987	4.2139	0.000584

Correlation coefficient R = 0.8875; Determination coefficient R^2^ = 0.7878; Adjusted determination coefficient R^2^adj = 0.7504; F test value F(3, 17) = 21.039; *p*-value < 0.00001; Std. error of estimation = 22.86.

**Table 5:** Regression model for ΔL/L_0_.

N = 21	Standardised Regression Coefficient Beta	Std. Error of Beta	Regression Coefficient b	Std. Error of b	*t* Test Value t(16)	*p*-Value
Intercept			−11.7418	0.8463	−13.8742	0.000000
% Cu	−0.0862	0.2366	−0.0060	0.0165	−0.3645	0.720280
% Sn	0.0167	0.2744	0.0064	0.1048	0.0607	0.952343
% Ni	0.4613	0.2623	0.0658	0.0374	1.7591	0.097660
% P	−0.1716	0.2963	−0.1334	0.2302	−0.5793	0.570442

Correlation coefficient R = 0.438866; Determination coefficient R^2^ = 0.1926; Adjusted determination coefficient R^2^adj = not applicable; F test value F(4, 16) = 0.9542; *p*-value < 0.45885; Std. error of estimation = 0.5154.

**Table 6:** Regression model for porosity.

N = 21	Standardised Regression Coefficient Beta	Std. Error of Beta	Regression Coefficient b	Std. Error of b	*t* Test Value t(16)	*p*-Value
Intercept			−0.0055	0.0212	−0.2590	0.798932
% Cu	−0.0289	0.2001	−0.0001	0.0004	−0.1446	0.886810
% Sn	−0.0667	0.2323	−0.0007	0.0026	−0.2870	0.777808
% Ni	−0.1991	0.2227	−0.0008	0.0009	−0.8941	0.384527
% P	0.7461	0.2514	0.0174	0.0058	2.9676	0.009071

Correlation coefficient R = 0.65191272; Determination coefficient R^2^ = 0.4250; Adjusted determination coefficient R^2^adj = 0.2812; F test value F(4, 16) = 2.9564; *p*-value < 0.5258; Std. error of estimation = 0.0129.

## Data Availability

The original contributions presented in this study are included in the article. Further inquiries can be directed to the corresponding author.
